# Supplementary material for: Kidney Allograft Monitoring by Combining Donor-Derived Cell-Free DNA and Molecular Gene Expression: A Clinical Management Perspective
Source: J Pers Med. 2023 Jul 29;13(8):1205. doi: 10.3390/jpm13081205 (PMC10455393; doi:10.3390/jpm13081205)
Supplement: Supplementary file 1 [file jpm-13-01205-s001.zip › jpm-2521780-supplementary.pdf]

Supplementary Materials:

Table S1. C4d positive antibody-mediated rejection (C+ABMR) vs. C4d negative antibody-mediated rejection (C-ABMR) groups

	**C4d+ve Antibody-Mediated Rejection (C+ABMR) Group (N = 11)**	**C4d-ve Antibody-Mediated Rejection (C-ABMR) Group (N = 17)**	**P-value**
**Donor-derived Cell-free DNA (dd-cfDNA)**			
dd-cfDNA pre-intervention Median (IQR)	2.80 (1.90, 4.65)	2.30 (0.88, 3.61)	0.38
dd-cfDNA post-intervention at 1 month Median (IQR)	1.16 (0.70, 4.80)	1.70 (0.32, 2.10)	0.79
dd-cfDNA post-intervention at 2 months Median (IQR)	1.70 (0.93, 3.45)	1.60 (0.92, 2.70)	0.84
dd-cfDNA post-intervention at last follow-up Median (IQR)	0.81 (0.52, 1.00)	1.35 (0.68, 2.12)	0.11
**Serum Creatinine (SCr)**			
SCr pre-intervention Median (IQR)	1.78 (1.54, 2.88)	2.50 (1.47, 3.91)	0.17
SCr post-intervention at 1 month Median (IQR)	1.95 (1.45, 2.75)	1.78 (1.31, 2.21)	0.28
SCr post-intervention at 2 months Median (IQR)	1.76 (1.40, 3.07)	1.55 (1.19, 2.40)	0.41
SCr post-intervention at last follow-up Median (IQR)	2.29 (1.73, 2.92)	1.47 (1.13, 2.53)	0.29
**Urine Protein-to-Creatinine Ratio (UPCR)**			
UPCR pre-intervention Median (IQR)	0.25 (0.14, 2.56)	0.53 (0.20, 2.08)	0.72
UPCR post-intervention at 1 month Median (IQR)	0.47 (0.37, 4.62)	0.37 (0.20, 1.61)	0.40

UPCR post-intervention at 2 months<br>Median (IQR)	0.76 (0.54, 3.11)	0.25 (0.17, 0.85)	0.15
UPCR post-intervention at last follow-up<br>Median (IQR)	0.26 (0.16, 0.88)	0.25 (0.14, 1.98)	0.64

*dd-cfDNA, donor-derived cell-free DNA; SCr, serum creatinine; UPCR, urine protein to creatinine ratio; IQR, interquartile range

Table S2. Antibody-mediated rejection with HLA DSA positivity (D+ABMR) vs. antibody-mediated rejection with HLA DSA negativity (D-ABMR) groups

	**Antibody-Mediated Rejection with HLA DSA Positivity (D+ABMR) Group (N = 15)**	**Antibody-Mediated Rejection with HLA DSA Negativity (D-ABMR) Group (N = 13)**	**P-value**
**Donor-derived Cell-free DNA (dd-cfDNA)**			
dd-cfDNA pre-intervention<br>Median (IQR)	2.50 (1.09, 3.81)	2.40 (0.90, 3.70)	0.73
dd-cfDNA post-intervention at 1 month<br>Median (IQR)	1.75 (0.82, 2.65)	0.74 (0.32, 1.90)	0.41
dd-cfDNA post-intervention at 2 months<br>Median (IQR)	1.60 (1.06, 2.85)	1.85 (0.67, 3.00)	0.80
dd-cfDNA post-intervention at last follow-up<br>Median (IQR)	1.15 (0.67, 2.07)	1.20 (0.48, 1.45)	0.65
**Serum Creatinine (SCr)**			
SCr pre-intervention<br>Median (IQR)	1.36 (1.15, 2.88)	1.54 (1.22, 1.77)	0.69
SCr post-intervention at 1 month<br>Median (IQR)	1.53 (1.20, 2.91)	1.42 (1.13, 2.06)	0.54
SCr post-intervention at 2 months	1.39 (1.17, 3.28)	1.41 (1.20, 1.92)	0.76

Median (IQR)			
SCr post-intervention at last follow-up<br>Median (IQR)	1.86 (1.31, 2.64)	1.51 (1.23, 2.56)	0.68
**Urine Protein-to-Creatinine Ratio (UPCR)**			
UPCR pre-intervention<br>Median (IQR)	1.18 (0.30, 4.20)	0.29 (0.14, 0.92)	0.09
UPCR post-intervention at 1 month<br>Median (IQR)	2.78 (0.47, 3.97)	0.30 (0.16, 0.71)	0.07
UPCR post-intervention at 2 months<br>Median (IQR)	0.66 (0.28, 1.80)	0.22 (0.20, 0.62)	0.16
UPCR post-intervention at last follow-up<br>Median (IQR)	0.51 (0.27, 1.19)	0.26 (0.16, 0.93)	0.37

*dd-cfDNA, donor-derived cell-free DNA; SCr, serum creatinine; UPCR, urine protein to creatinine ratio; IQR, interquartile range
